# Phase change thin films for non-volatile memory applications

**DOI:** 10.1039/c9na00366e

**Published:** 2019-09-18

**Authors:** A. Lotnyk, M. Behrens, B. Rauschenbach

**Affiliations:** Leibniz Institute of Surface Engineering (IOM) Permoserstr. 15 04318 Leipzig Germany andriy.lotnyk@iom-leipzig.de

## Abstract

The rapid development of Internet of Things devices requires real time processing of a huge amount of digital data, creating a new demand for computing technology. Phase change memory technology based on chalcogenide phase change materials meets many requirements of the emerging memory applications since it is fast, scalable and non-volatile. In addition, phase change memory offers multilevel data storage and can be applied both in neuro-inspired and all-photonic in-memory computing. Furthermore, phase change alloys represent an outstanding class of functional materials having a tremendous variety of industrially relevant characteristics and exceptional material properties. Many efforts have been devoted to understanding these properties with the particular aim to design universal memory. This paper reviews materials science aspects of chalcogenide-based phase change thin films relevant for non-volatile memory applications. Particular emphasis is put on local structure, control of disorder and its impact on material properties, order–disorder transitions and interfacial transformations.

## Abstract 2

The paper reviews materials science aspects of chalcogenide-based phase change thin films relevant for non-volatile memory applications.

## Graphical abstract

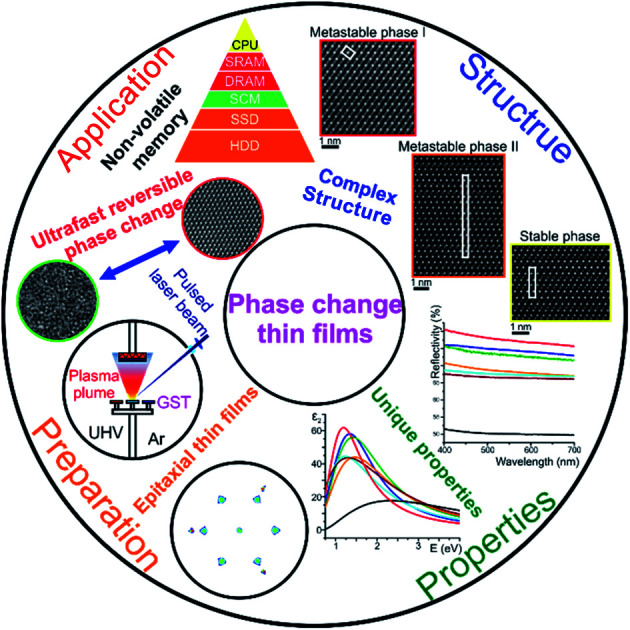

## 1. Introduction

**Fig. 1:**
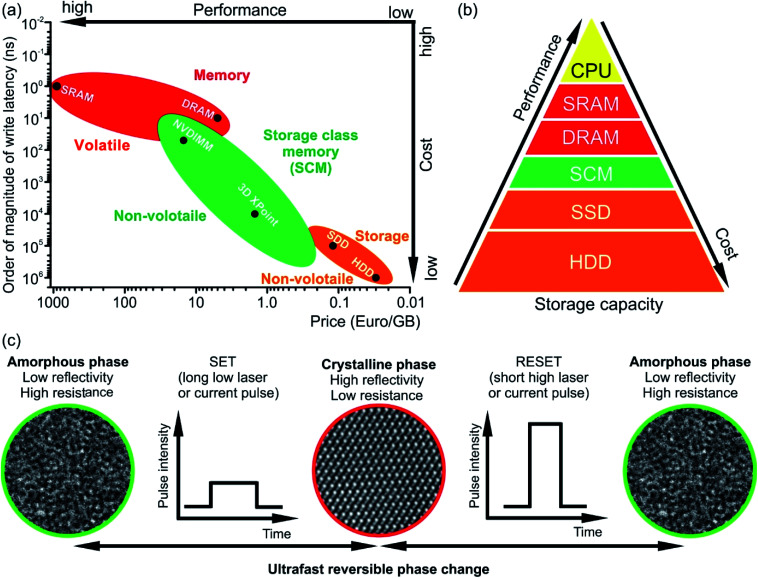
(a) Comparison of write latencies (order of magnitude) of different types of commercially available memories *versus* price per gigabyte (GB). The data are shown on the logarithmic scale. The accurate values of the latency can be found elsewhere.^6,28–34^ The estimated price is calculated using commercial memory products offered by online retailers. NVDIMM refers to the non-volatile dual in-line memory module. In the figure, the data for the module of NVDIMM type offering performance of DRAM are shown. (b) Memory hierarchy. The storage class memory (SCM) based on PCM can fill the gap in performance between the storage and memory. However, due to its high price, the NVDIMM (*e.g.* Intel Optane DC Persistent Memory) is mainly utilized for server memory, while the 3D XPoint memory (*e.g.* Intel Optane PCM) can be used in personal computers for acceleration of I/O operations. (c) The operation principle of PCM based on phase change materials. The structural phase of the material determines the logical state of a PCM cell. The crystalline phase is equivalent to state 1 while the amorphous phase is equivalent to state 0.

The rapid development and increasing number of Internet of Things devices require storage and on-line processing of a huge amount of data. The International Data Corporation predicts that the amount of processed data will grow from 44 zettabytes in 2020 to 175 zettabytes by 2025.^1^ The handling of such giant data volume requires, thus, fast and non-volatile memory technologies for both data processing and storage. The standard computing technology uses complex architectures and many interconnected components (*e.g.* processors (CPU), hard disks for data storage (Solid State Drive (SSD) or Hard Disk Drive (HDD)) and data transfer systems such as dynamic or static random access memory devices (DRAM and SRAM, respectively)). The speed of such systems during data-intensive tasks is limited by the characteristics of SDD or HDD devices since the data to be processed cannot be retrieved fast enough. In contrast, the emerging in-memory computing technology relies on the storage of information being processed in the main random access memory (RAM) of specialized servers rather than in complex databases utilizing slow hard disks. This technology offers the possibility for fast, real-time processing of a huge amount of data. However, RAM technology, *e.g.* DRAM and SRAM, has several significant limitations to deal with for application in next generation systems. For example, DRAM and SRAM are volatile memories and, thus require constant operating power. In addition, DRAM is difficult to scale up. Although the widely used NAND (NOT AND) Flash technology (*e.g.* SSD) in consumer devices is non-volatile, the Flash storage device is still slow (write/erase time ∼10^5^ ns compared to 10 ns for DRAM, Fig. 1(a)).^2^ On the other hand, phase change memory (PCM) technology based on chalcogenide phase change materials meets many requirements of the emerging data storage devices since it is fast, scalable and non-volatile. In addition, PCM offers multilevel data storage and can be applied both in neuro-inspired and all-photonic in-memory computing.^3–11^ PCM and other non-volatile memories such as resistive RAM (ReRAM) or magnetic RAM (MRAM) are regarded as storage class memory (SCM). The performance and cost of SCM is in between that of NAND flash and DRAM (Fig. 1(a) and (b)).^2,6^ Joint development of Intel and Micron resulted in the commercialisation of SCM in the year 2017 and the production of the Optane memory based on 3D XPoint technology.^12,13^ In this technology, Ge–Sb–Te-based materials are used as memory units for data storage.

The working principle of conventional PCM relies on ultrafast (ranging between 1 ps and several tens of ns) reversible phase transitions between crystalline and amorphous states of Ge–Sb–Te based materials (Fig. 1(c)).^14–18^ Originally, it was proposed by S. R. Ovshinsky in the late 1960s;^19,20^ PCM technology was first commercialized in optical storage media such as CD, then in DVD and finally Blu-ray disks. For data storage, PCM uses a large contrast either in electrical resistance between the amorphous state (logic state 0, high-resistance state) and crystalline state (logic state 1, low-resistances state) or in optical reflectivity between the amorphous state having low reflectivity and crystalline state possessing high reflectivity. The write process is realised by applying either electrical or optical pulses with low intensity, resulting in the crystallization of an amorphous bit at temperatures of ∼700 K.^21,22^ The erase process is accomplished by using a high intensity either electrical or laser pulse leading to the amorphization *via* melting and subsequent fast quenching of the phase change material.^23^ However, since phase change alloys are poor glass formers, rapid cooling rates are required to suppress the recrystallization of the alloy when it is to be amorphized.^17,24–27^ This can be achieved by appropriate device design. Moreover, the long-term stability of phase change materials is an issue and has to be improved by materials design.

Having such high potential for memory applications, the properties of different phase change alloys have been studied since their discovery. The contrast in the optical properties of phase change alloys can be explained by the differences in structural parameters (local order and distortions) and bonding mechanisms of the two phases.^35–37^ The origin of ultrafast crystal growth of phase change materials is attributed to the interplay between the structurally flexible building units of the amorphous matrix and fast crystal-growth kinetics.^38,39^ Many experimental studies are devoted to the study of order–disorder transitions in phase change materials.^40–44^ The amorphous and crystalline phases possess a similar local “distorted octahedral” environment of Ge and Sb, although the amorphous phase contains tetrahedral Ge units in addition.^42,45,46^ Moreover, distortions in the crystalline phase may trigger a collapse of long-range order too, producing the amorphous state without going through the liquid phase.^41^

Although the amorphous and crystalline phases of phase change materials possess some structural similarities, the chemical bonding of these phases is distinct. The atomic structure of the crystalline phase is stabilized by a unique type of bonding (in the past called resonant bonding while a new interpretation calls it metavalent bonding).^35,36,43,47–49^ This type of bonding is responsible for the high electronic polarizability and optical dielectric constants reported for crystalline phase change materials.^35,50^ However, this type of bonding requires long-range order. Since this order vanishes in the amorphous state, the phase shows an ordinary covalent type of bonding with lower polarizability.^37,51^ More interestingly, many phase change materials undergo order–disorder transitions within their crystalline phases.^52–56^ This is attributed to the presence of a huge amount of structural vacancies in the materials, which are also responsible for the p-type conductivity of phase change alloys.^57^ Depending on the composition, the number of structural vacancies in the cation sublattice can be up to 29% (*e.g.* for GeSb_4_Te_7_).^58,59^ In the crystalline phase the vacancies can be found randomly distributed or they can be ordered into vacancy layers. These several different arrangements indicate their important role in the formation of a variety of new transition structures that have a large impact on electronic and optical properties (*e.g.* charge transport, metal–insulator transition, and switching characteristics).^58,60,61^ The phases with ordered vacancies show metallic behaviour, while crystalline phases with disordered vacancies possess insulating behavior.^60–62^ Thus, control of disorder in phase change materials is very important for the application. Therefore, it is essential to reveal the local structure of relevant phases of the alloys. Moreover, nanoscale observation of atomic scale processes in such materials is also crucial for optimization of the performance of PCM and for understanding the underlying phase transitions in phase change materials.

This review focuses on materials science aspects of Ge–Sb–Te based phase change thin film materials, particularly focusing on atomic structure, impact of disorder on material properties, control of disorder, order–disorder transitions and interfacial transformations. The first part of the article describes the local structures of different phases of chalcogenide-based phase change alloys. The second part concentrates on the impact of disorder on material properties, including electrical conductivity, insulator–metal transition and optical reflectivity. The third part concerns precise control of disorder in phase change materials by growing epitaxial thin films of single-crystalline/phase quality with specific vacancy arrangement. The chemical disorder and structural defects in epitaxial thin films are also considered in that part. The fourth part discusses order–disorder transitions and interfacial transformations in Ge–Sb–Te materials with special emphasis on vacancy ordering processes, the cubic–trigonal phase transition, interlayer exchanges in layered Ge–Sb–Te crystal structures and ultrafast interface-controlled crystallisation processes in Ge–Sb–Te materials.

## 2. Local structure of chalcogenide-based phase change alloys

Among the Ge–Sb–Te (GST) based phase change alloys, Ge_2_Sb_2_Te_5_ (GST225) is a well-known and frequently studied alloy. GST225 is used as a model system for studying phase change behaviour and materials properties. While heating up the amorphous GST225 alloy, several temperature dependent phase transitions occur. GST225 and other similar ternary compounds on the pseudobinary GeTe–Sb_2_Te_3_ tie line first crystallize into several metastable structures and then at higher temperatures into a stable phase. Particularly, the metastable phases of Ge–Sb–Te alloys are important for data storage applications since these phases are formed during the write process (Fig. 1). The transition temperature is strongly dependent on the heating rate, interfaces and composition of the particular alloy.^26,63,64^

### 2.1 Metastable crystalline phases

**Fig. 2:**
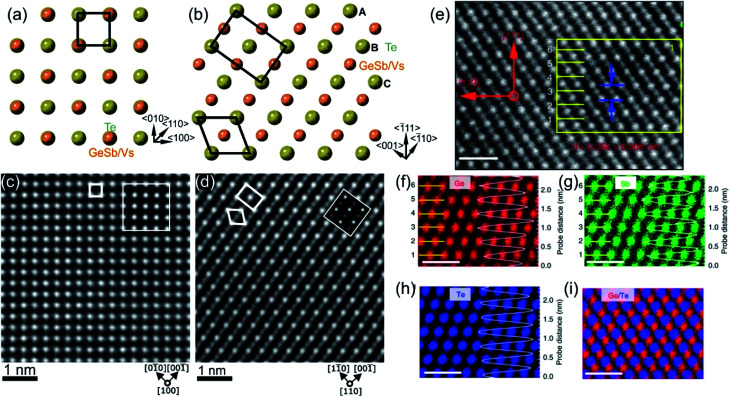
Crystal structure of GST225 phase I viewed along two crystallographic projections (a) 〈001〉 and (b) 〈110〉. Mixed GeSb/Vs atomic columns are arranged into layers in (b), whereas the columns are overlapped with Te columns in (a). The unit cells are marked by rectangles in (a) and (b). The main structural motif of GST225 phase I is marked in (b) by an octahedron. The ABC cubic stacking of the Te sublattice is also shown. (c) and (d) Experimental high-angle annular dark-field scanning transmission electron microscopy (HAADF-STEM) images of the GST225 crystal structure. The bright dots in (c) are Te/GeSb/Vs atomic columns. The bright dots in (d) are Te atomic columns whereas the darker dots are GeSb/Vs atomic columns. The insets show simulated HAADF images. (e) Atomic resolution energy dispersive X-ray spectroscopy (EDS) maps of GST phase I. Numbers 1–6 denote the cation layers. (f)–(h) Atomic resolution maps of Ge, Sb, and Te atoms taken from the marked area in (e), respectively. The EDS profiles along the [111] orientation are presented on the right-hand side showing uniform height peaks. (i) EDS maps of Ge/Te constructed by overlapping of (f) and (h). The maps show mixed Ge/Sb atomic positions. The scale bars are 1 nm in (e)–(i). The images (e)–(i) are reproduced from ref. 68, CC BY 4.0. Note: (c) and (d) Reprinted from Acta Materialia, 105, A. Lotnyk, S. Bernütz, X. Sun, U. Ross, M. Ehrhardt, B. Rauschenbach, Real space imaging of atomic arrangement and vacancy layers ordering in laser crystallized Ge_2_Sb_2_Te_5_ phase change thin film, 1–8,^52^ Copyright (2016), with permission from Elsevier.

**Fig. 3:**
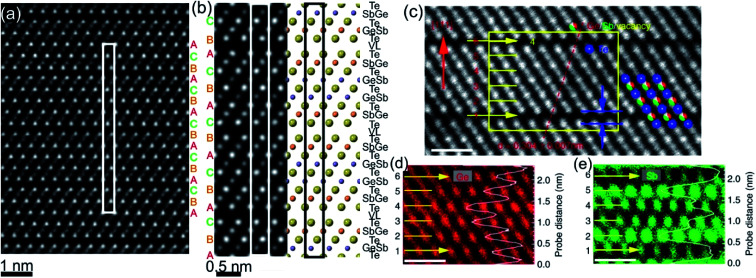
(a) Atomic-resolution HAADF-STEM image of GST225 phase II. The dark lines in the image represent vacancy layers (VLs). (b) Simulated HAADF image and structural model of phase II viewed along the [1120] direction. (c) HAADF image of GST phase II projected along the [110] orientation (using cubic settings). Numbers 1–6 denote the cation layers. (d) and (e) Atomic resolution EDS maps of Ge and Sb atomic columns. The EDS profiles along the [111] orientation are present on the right-hand side in every picture. The scale bars are 1 nm in (c)–(e). The maps suggest intermixed cation layers while the map in (e) show Sb-rich layers next to the VLs. The images (c)–(e) are reproduced from ref. 68, CC BY 4.0.

The first amorphous to crystalline phase transition is associated with the formation of the metastable crystalline GST225 phase (referred to as GST225 phase I). The crystal structure of phase I belongs to the cubic system and adopts a rock-salt structure (space group *Fm*3*m*, *a* = 0.6 nm).^59^ The anion sites in the cubic structure are occupied by Te atoms while the cation sites are randomly occupied by 40% Ge and 40% Sb as well as 20% vacancies (Vs), thus forming mixed GeSb/V sublattice sites (Fig. 2).^52,59^ Moreover, the Ge and Sb atoms are off-centred within idealized Te(GeSb)_6_ octahedrons. However, the degree of such distortions of the crystal lattice in the GST alloys depends on the amount of vacancies in the cation sublattices.^65^ GST materials with a high amount of vacancies show larger lattice distortions than compounds with a lower concentration of structural vacancies. The random distribution of structural vacancies within GST225 phase I is however energetically unfavourable. Upon thermal heating, the spatial distribution of vacancies in GST225 phase I changes since additional thermal energy supplies extra kinetic energy for atomic motions of Ge and Sb species. This leads to the rearrangement of Ge and Sb atoms and to gradual ordering of structural vacancies in the planes in the GST225 lattice.^53,55,66–68^ Therefore, different distributions of vacancies within the structure of GST225 result in different metastable GST225 structures, indicating polymorphism of metastable GST225 crystal structures. Finally, the most thermodynamically favourable GST225 phase contains a highly ordered distribution of vacancies in {111} planes (Fig. 3(a)). Theoretical calculations suggest this state as the lowest energetic state within ordered phases.^60^

The GST225 phase with highly ordered vacancy layers (referred to as GST225 phase II) consists of GST building blocks periodically separated by vacancy layers between adjacent Te–Te planes (Fig. 3(a) and (b)).^69^ Although the average chemical composition of phase II is close to the Ge_2_Sb_2_Te_5_ composition with 5 Te layers per building unit, in real samples the number of Te planes per building unit can vary from 4 to 6, showing local chemical disorder in such materials. As in the case of phase I, the Ge and Sb atoms are off-centre displaced within Te(GeSb)_6_ octahedrons. The Te–Te distances across the vacancy layers vary depending on the degree of vacancy ordering.^68^ The reported values for polycrystalline samples range between 0.33 nm (partially ordered) and 0.304 nm (fully ordered).^52,66–69^ However, GST-based materials of epitaxial thin films grown on a substrate show slightly larger values for the Te–Te distances (0.33–0.34 nm) as for relaxed polycrystalline thin films, probably either due to residual strain in the epitaxial films or due to thermodynamic stabilization effects towards the lowest energy structure.^69^ In all cases, the distances between Te planes across the vacancy layers are slightly larger compared to the Te–Te distances of 0.284 nm across the van der Waals gaps, representing similar vacancy layers within the stable GST225 phase (see the next subchapter). Considering all these structural aspects, partial filling of highly ordered vacancy layers by Ge atoms can be assumed in addition. The distribution of Ge and Sb atomic species is random within individual cation layers, showing mixed cation Ge/Sb layers similar to phase I (Fig. 3(c)–(e)).^68,69^ The preferential ordering of Sb atoms is however found in the cation layers next to the vacancy layers, whereas Ge species tend to accumulate preferentially in the middle of GST building units (Fig. 3). The formation of vacancy layers does not change the ABC-type cubic stacking of Te layers within the vacancy ordered phase. However, in order to incorporate these periodic layers into the cubic framework of phase II and considering solely the GST225 composition, the unit cell of phase II must be extended along the [111] direction to include 15 Te layers with three vacancy layers in between. Thus, phase II belongs to the trigonal structure (*P*3̄*m*1 space group) with unit cell parameters *a* = 0.426 nm and *c* = 5.126 nm. The crystal structure of phase II represents a superstructure of the rock-salt type with the cubic stacking of Te layers (ABC), in analogy to vacancy ordered chalcogenide alloys forming in the Ge–Sb–Te–Sn system.^54^

### 2.2 Stable crystalline phase

**Fig. 4:**
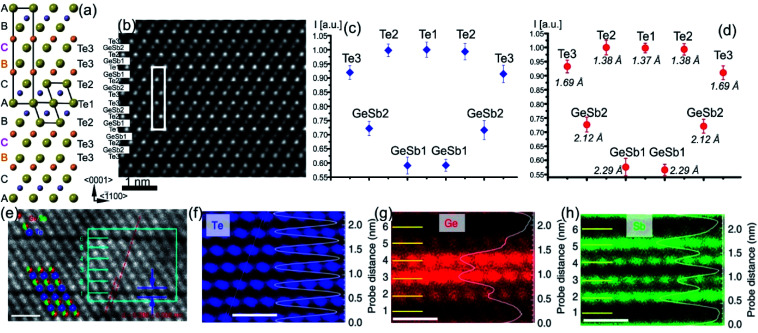
(a) Schematic representation of the trigonal GST225 crystal structure viewed along the [11–20] zone axis. The cations and anions form separate layers along this direction. The unit cell is marked by a rectangle. The main structural motif of the GST225 phase is marked by an octahedron. The stacking of the Te sublattice is also shown. (b) Atomic-resolution Cs-corrected HAADF-STEM image of the trigonal GST225 lattice in the [11–20] viewing direction. The unit cell is marked by a rectangle. The space between Te_3_ layers is the vdW gap. (c) Experimental average intensity maxima from Te and GeSb atomic columns extracted from the experimental image. (d) Normalized intensity maxima taken from simulated images using GST225 crystal structures reported in ref. 70. The numbers in the image are thermal displacement parameters. The simulated intensities are in good agreement with the experimental intensities. (e) HAADF image of the trigonal GST225 phase projected along the [11–20] orientation. Numbers 1 and 6 depict vdW gaps. Numbers 2–5 denote the cation layers. (f)–(h) EDS maps of Ge, Sb, and Te atomic columns. The scale bars are 1 nm. The map in (h) shows Sb-rich layers next to the vdW gaps while the map in (g) depicts Ge-rich layers in the middle of the GST225 building unit. The atomic resolution EDS maps confirm the results shown in (c) and (d). The images (e)–(h) are reproduced from ref. 68, CC BY 4.0.

The final phase transition during heating of metastable GST225 phases is accomplished with the formation of the stable GST225 structure. The stable GST225 phase belongs to the trigonal crystal system (*P*3̄*m*1 space group, *a* = 0.422 nm and *c* = 1.728 nm)^70^ and exhibits a layered structure with a 9P-type stacking sequence […–A–vdW–A–C–A–C–A–C–A–C–A–vdW–A–…] (a 9 layer cubic close-packed structure), where A refers to the anion, C refers to the cation and vdW refers to van der Waals gaps.^59,70^ The 9 layers make a building unit of the stable phase (Fig. 4(a)). These building blocks are stacked along the *c*-axis (using hexagonal settings) and are periodically separated from each other by vdW gaps, between adjacent Te layers. The stacking sequence of individual layers within each building unit is cubic. However, the cubic stacking is interrupted at the vdW gaps.

The structure of GST225 can be alternatively represented with overall composition (GeTe)*m*(Sb_2_Te_3_)*n*, where *m* building units of GeTe are interchanged with *n* units of Sb_2_Te_3_. The *m* = 2 and *n* = 1 result in the GST225 phase. The Ge and Sb atoms can be arranged into layers with different degrees of ordering within the structural unit of the stable trigonal GST225 phase. As a result, various structural models have been proposed for the trigonal GST225 phase.^59,70–72^ The models differ in the distribution of Ge and Sb atomic species at the cation sites, mainly. Theoretical calculations suggest ordered and disordered modifications of the trigonal phase, predicting either ordered or disordered arrangement of cation layers as the most energetically favourable structure of GST225.^73–76^ In contrast, the experimental X-ray and neutron diffraction studies as well as atomic-resolution transmission electron microscopy data show intermixed cation layers (Fig. 4(b)–(h)).^54,59,67,68,77–79^ The mixed Ge/Sb layers close to the vdW always are rich in Sb (*e.g.* Sb66Ge33 (ref. 70)) whereas the mixed cation layers in the middle of the GST building units are rich in Ge (*e.g.* Sb36Ge60 (ref. 70)), indicating some similarities to the local structure of GST225 phase II. Moreover, similar to the previous metastable GST225 phases, the cations in the trigonal GST225 structure are also displaced from the centre of the (GeSb)Te_6_ octahedrons. However, the distortions of (GeSb)Te_6_ octahedrons close to the vdW gap are stronger than the distortions of (GeSb)Te_6_ octahedrons in the middle of building blocks. The Te–vdW–Te layer distance is the most crucial parameter. It influences the band structure of the trigonal GST225 structure near the Fermi level. The enlargement of the *c*-parameter in the GST225 lattice from 1.725 nm to 1.85 nm causes a band-gap opening.^80^ This results in the elimination of the conducting interface states.

Overall, the crystal structures of crystalline GST225 phases are conceptually similar to each other. All crystal lattices are distorted and disordered on the cation sublattice, identifying, thus, the intrinsic structural properties of the GST225 phases.

## 3. Impact of disorder on material properties

### 3.1 Electrical conductivity and insulator–metal transition

**Fig. 5:**
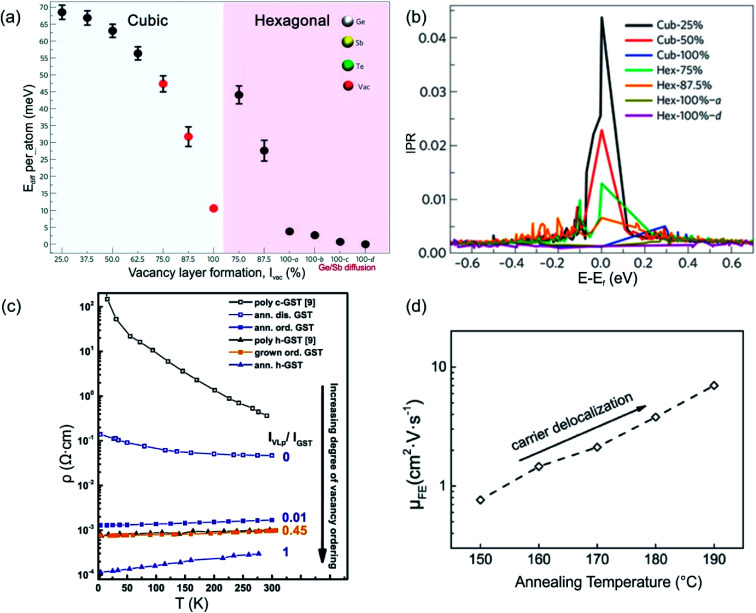
(a) Energy trend of GST phases with improvement of order in the cation sublattice. The models 100-a, 100-b, 100-c and 100-d refer to trigonal structures (marked by a hexagon in the image (a)) with different degrees of Sb/Ge mixing. (b) Inverse participation ratios (IPR) of the electronic states near *E*_f_ for the models shown in (a). For localized states, IPR remains finite and provides an estimate for the inverse of the number of atoms on which the state is localized. (c) Resistivity as a function of temperature for crystalline GST225 samples with different degrees of vacancy ordering. The number 0 means a fully vacancy disordered GST225 structure (phase I) whereas number 1 shows a vacancy ordered GST225 structure (stable trigonal phase). The number 0.01 shows measuring points for GST225 phase II. (d) Field effect mobility *versus* annealing temperature. The field effect mobility increases with increasing annealing temperature, indicating carrier delocalization in GST225. (a) and (b) Reproduced from ref. 62, CC BY 4.0. (c) and (d) Reproduced from ref. 61 and 88, respectively, CC BY 4.0.

Structural disorder (vacancies) has a huge impact on electronic and optical properties, *e.g.* charge transport and optical reflectivity, in crystalline GST materials.^58,61,81–83^ In GST225 phase I the vacancies are randomly distributed and are necessary to stabilize the structure as indicated by a negative formation energy.^65^ For example, the creation of vacancies in the Ge_2_Sb_2_Te_4_ compound reduces the average number of valence electrons and thus, the occupation of antibonding states.^65^ This finally results in the more stable compound. Although the Ge_2_Sb_2_Te_4_ phase cannot be prepared chemically,^84^ it serves as an example of the influence of vacancies on the overall stability of Ge–Sb–Te compounds. Moreover, lattice distortions further contribute to an additional strong decrease in energy, stabilizing the alloy. The octahedral-like arrangement of Ge, Sb and vacancies has been attributed to the formation of p-bonds between the atoms. Such p-bonds are very directional and are prone to local distortions, known as Peierls distortions.^85^ The high degree of structural disorder in cubic phase I leads to large electron scattering and, as a consequence, to electron low mobility, thus resulting in insulating behaviour.^58,61,82^ The combination of directional bonding, lattice distortions and high vacancy concentrations causes charge carrier localization.^60^ The microscopic origin of the electron localization was directly attributed to the presence of vacancy clusters on cation sites, which have a high impact on the nature of the wave functions. Density functional theory (DFT) calculations show that gradually improving structural order in the cation sublattice in GST materials by subsequent ordering of vacancies into vacancy layers leads to the vanishing of localized states and to a transition from an insulating to a metallic state (Fig. 5(a) and (b)). Hence, the metal–insulator transition (MIT) is a disorder-induced MIT and regarded as the Anderson MIT.^86,87^

The ordering of vacancies into vacancy layers results in the reduction of the total energy per atom (of the order of 50 meV per atom), leading to an energetically more favourable structure compared to those structures with random distribution of vacancies (Fig. 5(a)).^60^ Moreover, the energy differences between vacancy ordered GST phase II and the stable trigonal phase are small (of the order of 5–7 meV per atom). The small energy difference suggests that GST structures with ordered vacancies (phase II) can be produced under appropriate conditions and in the material state (*e.g.* as thin films).

Experimental evidence of the impact of disorder on the electronic properties of GST alloys is given in Fig. 5(c) and (d). Temperature dependent Hall measurements (Fig. 5(c)) show a positive temperature coefficient of conductivity (slope) for vacancy ordered GST phase II.^61^ This is an indication of metallic behaviour. On the other hand, the slope of the temperature coefficient for the vacancy disordered GST structure (phase I) is negative, pointing out to a non-metallic behaviour. The differences in the electrical resistivity of different phases are also evident from Fig. 5(c). Furthermore, field effect measurements can also provide useful information regarding localized states of carriers in semiconductors. For example, measurements on the GST225 alloy are presented in Fig. 5(d). The field effect mobility increases with increasing annealing temperature of the GST225 alloy.^88^ Since the increase in mobility is mainly due to the proportional reduction of localized carriers, the ordering of vacancies in GST alloys results in the delocalization of carriers, confirming theoretical predictions well.

The structural order in crystalline Ge–Sb–Te materials also correlates with different resistance states.^61,81,89^ This enables another route to realize multi-level data storage. Since structural relaxation of the amorphous phase causes a resistance drift,^90,91^ the multilevel storage devices based on crystalline structures will benefit from more stable resistance states over time.

### 3.2 Optical reflectivity

**Fig. 6:**
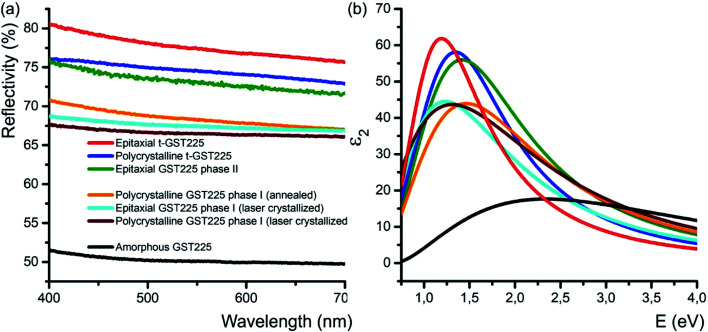
(a) Reflectivity of different GST225 thin films related to spectralon used as standard during the measurements. A reflectivity contrast of up to 29% between the amorphous GST225 film and the epitaxial t-GST225 thin film is obtained. Within the crystalline phases of GST225 the optical contrast is up to 12%. Reproduced from ref. 93 with permission from the Royal Society of Chemistry. (b) Imaginary part of the dielectric function of GST225 thin films shown in (a). A pronounced difference between the disordered and ordered phases is found. The colours in (b) refer to the same phase as shown in (a).

In addition to the electrical conductivity, spatial distribution of vacancies in GST materials influences the optical properties of the alloys. In particular, DFT simulations, and ellipsometric and optical reflectivity measurements reveal an increased optical reflectivity of vacancy ordered phases (GST225 phase II and the trigonal phase) compared to vacancy disordered GST225 phase I (Fig. 6(a)).^50,92,93^ These differences in optical reflectivity can be explained by changes in the free carrier absorption due to electron localization effects. Fig. 6(b) shows the imaginary part of dielectric function (*ε*_2_) for different GST225 phases. The *ε*_2_ characterizes the absorption of light and thus allows an assessment of the optical contrast. In accordance with the reflectivity measurements (Fig. 6(a)), the vacancy ordered phases (phase II and the trigonal phase) show higher *ε*_2_ compared to the disordered phases. At first glance, this effect could be attributed to an increase in carrier concentration. However, as electrical measurements in GST alloys show that the carrier concentration in these materials is only slightly increased from disordered phase I to the vacancy ordered phase,^81^ a more likely reason for the higher absorption is hence the increased carrier mobility due to vacancy ordering. The latter induces a delocalization of the electronic wave functions.^60^ As a result, the different crystalline phases exhibit a sufficient contrast in reflectivity for applications in optical data storage. Additionally, as shown in Fig. 6(a) doping of GST thin films with Sn as well as substitution of Te with Se have an enhancing effect on the optical contrast.^94,95^

Overall, by tuning the degree of structural order regarding the vacancy distribution and the associated degree of electron localization as well as texture of GST phase change alloys, distinct differences in optical and electrical properties within GST alloys can be achieved. Consequently, a proper engineering of disorder in different GST phases might enable the realization of multilevel resistance/reflectivity states accomplished by crystalline-to-crystalline phase changes, resulting in a drastic increase of the data storage density and reduction in the energy consumption of working devices, respectively.

## 4. Control of disorder and defects in crystalline phase change materials

### 4.1 Material growth

The choice of the physical deposition method for the production of GST thin films has to be related to the GST material, desired properties and application. The relevant methods are primarily magnetron sputtering, molecular beam epitaxy (MBE) and pulsed laser deposition (PLD). Many specialized books and overview articles cover each of these general vapour deposition methods and a detailed discussion lies outside the scope of this review.

The magnetron sputtering technique is based on the bombardment of a target (cathode) with noble gas ions situated in front of this target.^96^ The bombardment process causes the removal (*i.e.* sputtering) of the target atoms that may then condense on a substrate as a thin film. Secondary electrons, which play an important role, are also emitted from the target surface. Magnetrons are applied to overcome the limitations of this simple sputtering process. Thereby a magnetic field is configured parallel to the target surface to constrain secondary electron motion to the vicinity of the target. One pole of the magnets is positioned at the central axis of the target and the second pole is formed by a ring of magnets around the outer edge of the target. Then the trapped electrons increase the ionization probability of electron–gas atom collisions, where also, the probability of sputtering processes is significantly increased and, therefore, higher deposition rates can be accomplished.

The operating principle of MBE is based on the heat up of a source material in the so-called effusion cell to a high enough temperature where a considerable vapour pressure is realized.^97^ Then, the vapour molecules in the effusion cell reside in the thermodynamic equilibrium. *Via* a small hole in the wall of the cell, molecules will effuse out and form a molecular beam. Thereby the size of the hole is smaller than the average mean free path of the gas molecules. Consequently, the MBE method is characterized by a very small deposition rate and an energy of the deposited molecules of some tenths of electron volt.

**Fig. 7:**
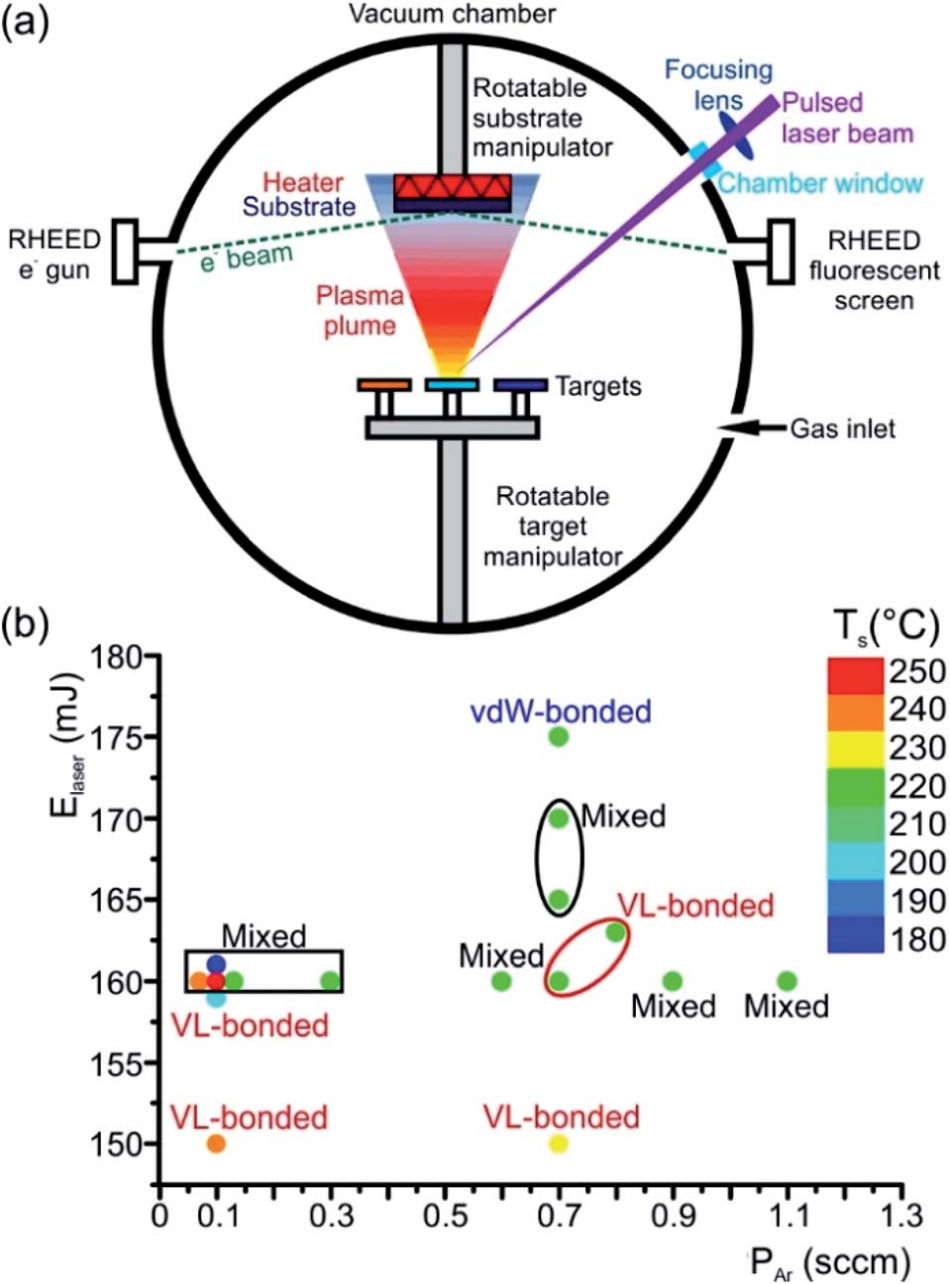
(a) Schematic representation of a PLD system. The ablated atomic species from a target surface form a plasma plume, which then condenses onto a substrate in the form of a thin layer. The process of thin film growth can be monitored *in situ* by using a RHEED system. (b) Phase map showing the influence of the laser energy *E*_laser_, partial pressure *P*_Ar_ of Ar background gas and substrate temperature *T*_s_ on phase formation during epitaxial GST225 thin film growth onto the Si(111) substrate. The vdW-bonded phase refers to the trigonal GST225 thin films whereas VL-bonded and mixed phases show GST225 phase II and a mixture of three different phases (phases I, II and trigonal GST225), respectively. The distance between the substrate and GST225 target was set to about 6 cm and the repetition rate was kept 2 Hz. For thin film deposition, a GST compound target with a GST225 stoichiometric composition and a KrF excimer laser with 248 nm wavelength and 20 ns pulse duration were used. The base pressure of the deposition chamber was 1.3 × 10^−8^ mbar.

An elegant method to deposit high-quality epitaxial thin films is the PLD (Fig. 7(a)), where any kind of material can be evaporated. This flexible and powerful physical vapour deposition technique is a growth technique in which the photon energy, the pulse duration and the laser wavelength are the key parameters for the interaction of the laser with a target material. As a result, the material is removed from the target depending on the absorption properties of the target material. In principle, a pulsed laser beam is focused onto the surface of a target in a vacuum chamber. A significant fraction of material is removed above a certain threshold power density and the ejected, partially ionized material forms the so-called plasma plume. The ablated material is directed towards a substrate where it condenses to generate a thin layer. Material species, growth temperature, type of the substrate material, pressure in the ablation chamber, the laser deposition parameters (laser energy, repetition rate, and pulse duration) and background gas influence the growth kinetics of the film, thus influencing phase formation in the as-deposited thin film (Fig. 7(b)). In the past, comprehensive overviews about the pulsed laser deposition have been published.^98,99^

Typically, pulsed laser deposition is carried out in an ultra-high vacuum chamber (Fig. 1(a)), operating at a base pressure in the range of 10^−8^ mbar. Additionally, the process can also occur in the presence of a background gas. For the deposition of chalcogenide epitaxial films an ∼5 × 10^−5^ mbar Ar partial pressure is employed by introducing Ar gas. The use of this Ar background serves the purpose to moderate the kinetic energy of particles in the plasma plume. Typically, KrF excimer lasers are used with a wavelength of 248 nm and a pulse duration between one and a few tens of nanoseconds. The laser beam is focused, *via* lenses through a quartz window, onto the surface of the rotating target at an incident angle of particularly 60° (in general between 50° and 75°)) with respect to the target normal. The pulse energy is varied between 10 and 200 mJ and the fluence ranges between 0.1 and 5 J cm^−2^ The pulse repetition rate can be varied between 1 and 100 Hz. For example, deposition rates up to 300 nm min^−1^ were possible at 100 Hz and 1 J cm^−2^.^100,101^

Stoichiometric sintered targets of different phase change materials are used. In general, these target materials possess an optical absorption coefficient of 10^6^ cm^−1^ in the utilized UV wavelength regime and are therefore suitable for the PLD process.^99^ After a cleaning procedure prior to deposition, the substrates are positioned inside the vacuum chamber parallel to the target surface (Fig. 7(a)). Both target and substrates are rotated in order to suppress deep ablation of the target and to avoid inhomogeneity in the thickness of thin films. A resistive heater source is mounted in a specific position on the substrate holder, near the substrates, in order to ensure an effective thermal dissipation when increasing the substrate temperature to values required for the experiments (temperatures between room temperature and some hundred degree Celsius). The substrate heater mount is calibrated by thermocouple measurements on various substrate materials. Depending on the laser fluences (laser pulse energy per laser spot area) extreme local heating and consequently desperate cooling rates can be reached that cause the ablation of the material, by which the material is removed from the irradiated substrate. Then, the material being ablated is expelled from the surface with a highly forward directed species distribution, the so called plasma plume. The distribution of the material ablated from the target surface is symmetrically peaked along the normal axis of the surface and falls-off as (cos *θ*)^*q*^, where *θ* is an angle diverged from the normal axis and *q* usually ranges between ∼4 and 30.^98,102^

The plasma plume consists of a number of different species, involving electrons, ions, neutrals, and clusters. For the ablation of Ge_2_Sb_2_Te_5_, the plasma plume consists of Ge^+^, Ge_2_^+^, GeTe, Te_2_^+^, GeTe_2_^+^, overlapped clusters of (SbTe_2_, Sb_2_Te and Te_3_), GeSbTe_2_^+^, and Sb_3_Te^+^, and low intensity GeSbTe_3_^+^ and Sb_2_Te_3_.^103^

Often different surface and thin film analysis techniques are integrated into the deposition chamber. As an example, a reflection high energy electron diffraction (RHEED) system is installed (Fig. 7(a)). RHEED allows *in situ* monitoring of the surface crystallinity and smoothness of layers during deposition. Moreover, low-angle X-ray spectroscopy (LAXS) can be used during the thin film growth process for *in situ* real time composition analysis.

### 4.2 Epitaxial phase change thin films with different vacancy structures

The formation of a particular phase during the growth of GST thin films depends strongly on the deposition process parameters that influence relevant quantities like the momentum and kinetic energy of impinging species or their diffusivity on the substrate surface (Fig. 7(b)). A key process parameter is the temperature of the substrate during thin film growth, which provides a sensible phase selectivity. In the case of a substrate at room temperature, the resulting GST thin films obey an amorphous, disordered structure, whereas at elevated temperatures crystalline growth is possible. Here, however, the substrate temperature must not necessarily equal or exceed the crystallization temperature of the growing material, since techniques like sputter deposition and PLD provide particles which deliver kinetic energies of up to hundreds of eV enabling crystalline growth at lower temperatures.^104^ Reports demonstrating the low temperature epitaxy of GST thin films by PLD and magnetron sputter deposition can be found in ref. 82 and 104, respectively. Another important aspect apart from the substrate temperature is its atomic structure, which affects the diffusion barrier of the adsorbed atoms. Moreover, if the surface is crystalline and has a low lattice mismatch with respect to the GST thin film, epitaxial growth becomes possible. However, it should be noted, that in the case of Sb_2_Te_3_ and GeTe–Sb_2_Te_3_ superlattices a tendency of textured growth is also observed on amorphous substrates, which could be ascribed to the layered nature of these materials and weak coupling between building units.^105–107^

Epitaxial GST thin films with a disordered vacancy structure can be grown by MBE on (001) oriented GaSb substrates and they obey a simple cube-on-cube epitaxial relationship.^108^ Subsequent studies, however, revealed that epitaxial GST thin films grown on (111) oriented GaSb and InAs substrates exhibit superior properties in terms of both surface smoothness and crystallinity. This is attributed to a tendency of GST to form (111) oriented layered structures.^109,110^ Similar results were found for PLD grown epitaxial GST thin films on BaF_2_(111) in a temperature window ranging between 85 °C and 295 °C.^104^ In addition to MBE and PLD methods, the fabrication of GST thin films with different vacancy structures on freshly cleaved muscovite mica substrates is also possible by DC magnetron sputtering.^82^

**Fig. 8:**
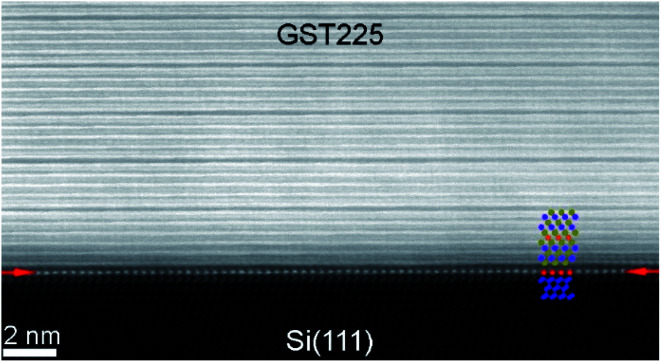
Atomic-resolution HAADF-HRSTEM image of the interface between the Si(111) substrate and trigonal GST225 thin film. The Sb/Te passivation layer on top of the substrate is marked by red arrows. The insert shows a scheme of the Si–Sb/T-GST225 interface. The Si atoms are marked by blue spheres and the Sb/Te passivation layer is shown by red spheres whereas Te atoms are marked by olive spheres and azure/orange spheres represent Sb/Ge atoms. Due to the slight in-plane rotation of the GST225 crystal structure, the Te, Sb and Ge atomic columns are not resolved in the HAADF image.

A substrate proven to be suitable as well as technologically relevant for the epitaxial growth of GST thin films is Si(111).^61,101^ Although there is a large lattice mismatch of more than 10% between GST and Si, well oriented and epitaxial thin films with full width at half maximum values of XRD rocking measurements of 0.05° were demonstrated.^78,101^ Hence, the lattice matching condition can be bypassed, which is attributed to a special case of epitaxy, so called vdW epitaxy.^111^ Here, only weak interactions between the substrate and the thin film are present and allow the growth of highly lattice mismatched epitaxial heterostructures.^78^Fig. 8 depicts a detailed HAADF-HRSTEM image showing the vdW epitaxy in the case of GST225 on Si(111). Upon closer inspection of the interface, a thin layer of Sb/Te atoms passivating Si dangling bonds at the substrate surface is observed. This passivation layer is formed during the initial stages of deposition followed by the emergence of a vdW gap bridge from the passivation layer to the first Te layer of the growing GST thin film.^78,112,113^ Consequently, only weak interactions between the substrate and thin film are present enabling almost strain-free epitaxy. The strain energy between the substrate and thin film is released first by introduction of misfit dislocation into the passivation layer and second by slight in-plane rotation of GST grains due to domain-matched epitaxy (Fig. 8).^69,78,114–119^ Thus, this type of epitaxy is common for chalcogenide based phase change materials.

Recent advances in the epitaxial growth of GST based thin films on Si(111) by MBE and PLD have provided deeper insights into the microstructure evolution in metastable cubic GST phase I induced by a vacancy ordering phenomenon. In general, by gradually increasing the substrate temperature during the growth process, the crystalline quality of initially cubic GST thin films can be improved. At the same time, however, this leads to the formation of GST structures with a higher degree of structural order, which primarily manifests in vacancy layer formation accompanied by a decrease in compositional randomness on the cation layers due to a partially ordered stacking sequence.^68,77^ By supplying even more energy to the growth process, the stable trigonal phase of GST can be grown. Moreover, since there is only a thin line separating the formation of different crystalline phases during epitaxy, a mixed growth of stable and metastable GST with heterogeneous vacancy structures frequently occurs.^69^ This is due to the close formation energies of different vacancy ordered GST phases.

The formation of regularly spaced vacancy layers in epitaxially grown GST thin films (phase II) is associated with the occurrence of additional diffraction peaks in XRD *θ*–2*θ* measurements compared to the expected XRD pattern of GST thin films containing phase I.^61,93^ These additional diffraction peaks can be regarded as superstructure reflections due to the vacancy layer substructure within the (pseudo)cubic lattice. In general, the resulting crystal structure becomes closely related to that of the stable trigonal phase of GST, which yields only minor differences in 2*θ* values of the corresponding XRD diffractograms. Therefore, it is crucial to reliably distinguish between the two phases if the vacancy ordering phenomenon is to be studied independently of a structural phase transition towards the stable trigonal phase.

**Fig. 9:**
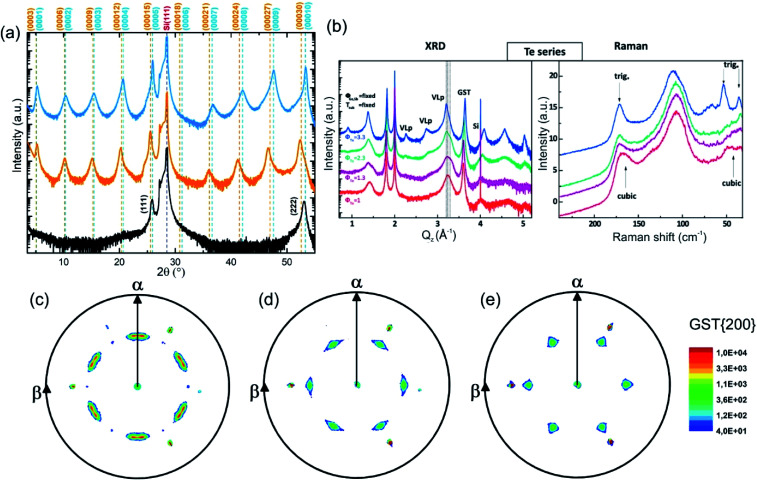
(a) *θ*–2*θ* XRD measurements of an epitaxial t-GST225 thin film (black curve), an epitaxial GST225 phase II film (red curve) and an epitaxial c-GST225 thin film (green curve). The thin films were grown by PLD. Calculated peak positions of t-GST225 using lattice parameters reported by Matsunaga *et al.* are indicated by black vertical lines.^59^ For the calculation of GST225 phase II reflections a unit cell with the lattice parameters *a* = 0.426 nm and *c* = 5.221 nm and assumed to have a trigonal space group (*P*3̄*m*1) was used. (b) XRD and Raman spectra of GST samples grown at different Te fluxes (*ϕ*_Te_) by MBE. The red curve (the same sample as in the XRD plot) in the Raman spectra is in accordance with phase I, while the sample grown at the highest Te flux is a trigonal thin film. The intermediate curves can be considered as being due to a mixture of different phases. Reprinted from ref. 120, with the permission of AIP Publishing. (c)–(e) In-plane XRD pole figures of the GST225(200) reflections of: (c) an as-deposited epitaxial stable GST225 thin film, (d) an as-deposited epitaxial metastable GST225 thin film with vacancy ordering and (e) a laser crystallized epitaxial metastable GST225 film without vacancy ordering. The samples were deposited by PLD. Slight in-plane rotation of GST225 domains is obvious in (c) and (d). Reproduced from ref. 93 with permission from the Royal Society of Chemistry.

The growth of single-phase highly ordered GST225 phase II and stable epitaxial GST225 thin films on Si(111) substrates can be realised by PLD (Fig. 9(a)) and MBE (Fig. 9(b)).^61,120,121^Fig. 9(a) depicts three exemplary XRD diffraction patterns of epitaxial GST225 thin films grown on Si(111) by PLD. Although there are only small differences between the first two XRD patterns, they originate from GST225 thin films of different phases: the stable trigonal phase (black curve) and the metastable vacancy ordered phase of GST225 (red curve). Accordingly, the differences in Bragg reflections can be associated with the differences in superstructure layering between the two phases. In contrast, the black curve in Fig. 9(a) featuring only the cubic GST225(111) and cubic GST(222) reflections corresponds to an epitaxial cubic GST thin film which is characterized by random distribution of vacancies (phase I) and thus no superstructure reflections appear. This phase can be obtained in an elegant way by ns-laser crystallization of amorphous GST thin films deposited on crystalline Si(111) substrates or by laser induced recrystallization of trigonal GST225 thin films,^83,93^ while GST225 phase II can be produced by fs-laser irradiation.^61^ The fabrication process of phase I ensures that no vacancy ordering is present, whereas during preparation methods involving thermal annealing on longer timescales partial vacancy ordering might occur.

Based on these results, the out-of-plane epitaxial relationships of the GST thin films can be specified: c-GST(111)‖Si(111), phase II GST(0001)‖Si(111) and t-GST(0001)‖Si(111). Information on the in-plane orientation of the thin films can be obtained from the corresponding XRD pole figure measurements which are presented in Fig. 9(c)–(e): c-GST[11-2]‖Si[11-2], phase II GST225[11-20]‖Si[11-2] and t-GST225[11-20]‖Si[11-2]. Moreover, the pole figures verify the presence of 180° rotational twin domains indicated by the occurrence of two sets of three GST pole density maxima in each pole figure. This twinning is regularly observed in epitaxial GST thin films and its degree can be controlled by employing Si(111) miscut substrates during epitaxy, which offers a route to material engineering (*e.g.* tuning of the electronic band structure).^121,122^

Overall, state-of-the-art deposition techniques allow the growth of high-quality GST based thin films with different degrees of disorder on different substrates. Since the synthesis of bulk single crystals of GST225 phases I and II has not been realized to date, single phase epitaxial thin film systems offer a considerable potential for basic research^61,82,123^ and the development of new concepts of memory devices in the future.

### 4.3 Chemical disorder and structural defects

**Fig. 10:**
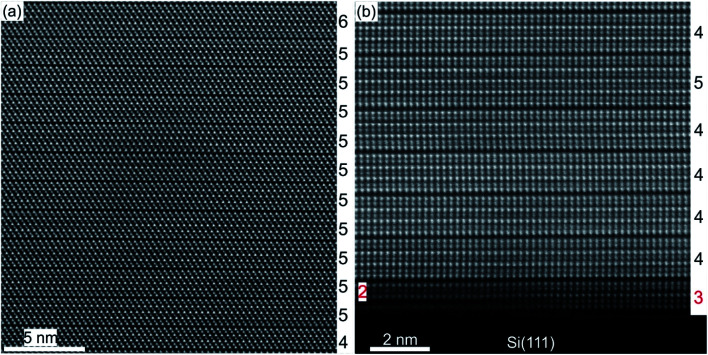
(a) HAADF-HRSTEM image of t-GST225 showing the presence of GST225, GST124 and GST236 building blocks. Viewing direction is 〈11–20〉. (b) Atomic-resolution HAADF image of the GST225 thin film deposited at 280 °C. Due to the change in composition during thin film growth, Te stacking is predominantly 4-fold (GST124). In addition, the formation of 5Te (GST225), 2Te (GeTe_2_) and 3Te (Sb_2_Te_3_) stacking units is visible in the micrograph. Viewing direction is 〈1–100〉. The numbers in (a) and (b) indicate the local stacking of Te layers. Notes: (a) Reprinted from Materials & Design, 115, I. Hilmi, A. Lotnyk, J. W. Gerlach, P. Schumacher. B. Rauschenbach, Epitaxial formation of cubic and trigonal Ge–Sb–Te thin films with heterogeneous vacancy structures, 138–146,^69^ Copyright (2017), with permission from Elsevier.

The as-grown epitaxial GST225 thin films with a trigonal structure usually possess local chemical disorder. While on average the building unit contains (separated by vdW gaps) 5 Te layers, the structural units with 4 and 6 Te planes are also formed in equal amounts.^69,72,78,124^ These units correspond to trigonal GeSb_2_Te_4_ (GST124) and Ge_3_Sb_2_Te_6_ phases. The formation of these phases is necessary to preserve the composition of the GST225 phase.^72,124^Fig. 10(a) gives an exemplary HAADF-STEM image of the epitaxial GST225 thin film grown on the Si(111) substrate.^69^ Contrary to the GST225 thin films, the trigonal GST124 thin films are not prone to chemical disorder.^125^ However, increased substrate temperature during deposition of GST225 thin films can lead to preferential evaporation of Ge from the GST225 compound. This might result in the preferential formation of the GST124 phase as well as Sb_2_Te_3_ and GeTe_2_ building units (Fig. 10(b)).^44,78^ While the trigonal Sb_2_Te_3_ structure is a well-known compound, the layered GeTe_2_ material forms a layered structure and is characterized by distorted tetrahedral coordination of Ge. DFT calculations show the stabilization of freestanding GeTe_2_ under tensile strain and predicts a metallic behaviour for the compound.^126^ Thus, by appropriate selection of deposition parameters, a thin layer of GeTe_2_ can be in principle grown on the Si(111) substrate.

**Fig. 11:**
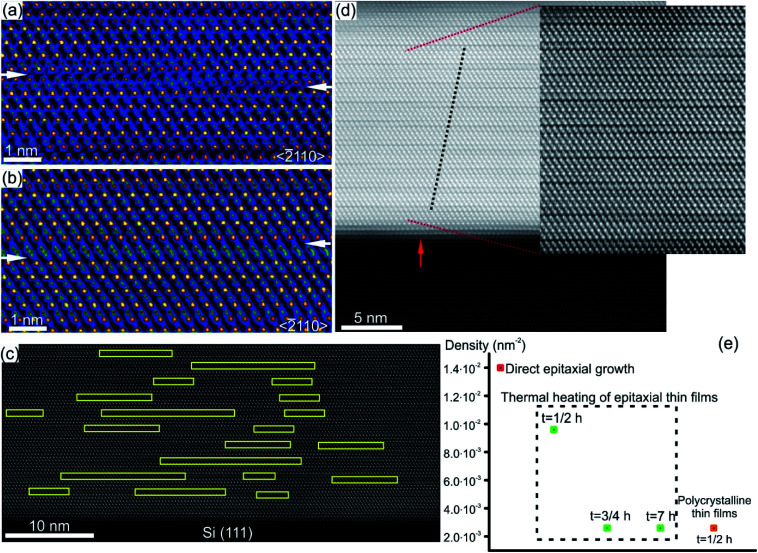
(a) and (b) Atomic-resolution HAADF-HRSTEM images (RGB) of bilayer defects in GST225 crystal structures seen along the 〈11–20〉 viewing direction. In incoherent imaging (Z-contrast), the intense dots correspond to Te atomic columns whereas the less intense dots represent GeSb atomic columns. Dark lines result from vdW gaps. GST building blocks with 4Te, 5Te and 6Te layers correspond to GST124, GST225 and GST326 crystal structures, respectively. The transition between Te layers in (a). The arrows mark the position of vdW gaps along the bilayer defects. (c) Spatial distribution of bilayer defects. (d) High-resolution HAADF-STEM micrograph of the GST/Si interface from the sample deposited at 280 °C. Due to the change in composition, Te stacking is predominantly 4-fold. The bilayer defects partially correlate (as indicated by dashed lines) with single-layer surface steps in the Si substrate (indicated by an arrow). (e) Density of bilayer defects in GST225 thin films with different textures and processing parameters. Notes: (a) and (b) Reprinted from Acta Materialia, 141, A. Lotnyk, U. Ross, T. Dankwort, I. Hilmi, L. Kienle, B. Rauschenbach, Atomic structure and dynamic reconfiguration of layered defects in van der Waals layered Ge–Sb–Te based materials, 92–96,^127^ Copyright (2017), with permission from Elsevier. (e) Reprinted from Journal of Alloys and Compounds, 676, U. Ross, A. Lotnyk, E. Thelander, B. Rauschenbach, Microstructure evolution in pulsed laser deposited epitaxial Ge–Sb–Te chalcogenide thin films, 582–590.^78^ Copyright (2016), with permission from Elsevier.

Characteristic structural defects in layered GST based and Sb–Te based crystal structures include rotational twin domains and particular defect structures, known as Te(GeSb) bilayer defects.^78,127–129^ The latter are formed by local reorganization of the stacking sequence across the vdW gaps connecting GST building blocks of different compositions. In this way, a GST225 block may change into a GST124 unit or GST326 block above or below (Fig. 11), appearing as interwoven into each other.^74,77,78,127,128^ This leads to the intrinsic changes of the height of GST building blocks on the left and right side of the transition area. Fig. 11(a) and (b) show the exemplary atomic-resolution HAADF-STEM image of such Te(GeSb) bilayer defects. The bilayer defects are frequently observed in epitaxial GST225 thin films (Fig. 11(c) and (d)). The density of these defects is, however, much lower in polycrystalline GST225 thin films (Fig. 11(e)). The bilayers defects are stable, mobile and possess the capability of self-reorganization throughout the film. Post-thermal heating of epitaxial GST225 thin films containing such defects for a prolonged time did not result in the annihilation of these bilayer defects (Fig. 11(e)). The confirmation on the defect mobility and self-reconfiguration at elevated temperatures in such layered GST materials was indeed done by *in situ* electron microscopy observations.^55,127,130^ The bilayer defects do not form at particular sites in the thin film. The defects are observed at various locations of GST225 thin films and at the interface between the film and the Si(111) substrate (Fig. 11(c)).^78^ The latter is due to monolayer surface step imperfections of the substrate (Fig. 11(d)). These particular bilayer defects nevertheless disappear within several nanometre distances from the interface which is due to strain release in the film and the equilibration of the local stoichiometry during GST225 thin film growth. Thus, the bilayer defects in GST thin films are formed during thin film deposition. DFT calculations explain the formation of a high-density of bilayer defects by low energy consumption (∼9.8 meV per atom) with respect to the unfaulty structure.^131^ Moreover, first-principles calculations and electron counting model analysis show that bilayer defects can influence the carrier concentration close to the Fermi level, promoting a metal–insulator transition.^132^

**Fig. 12:**
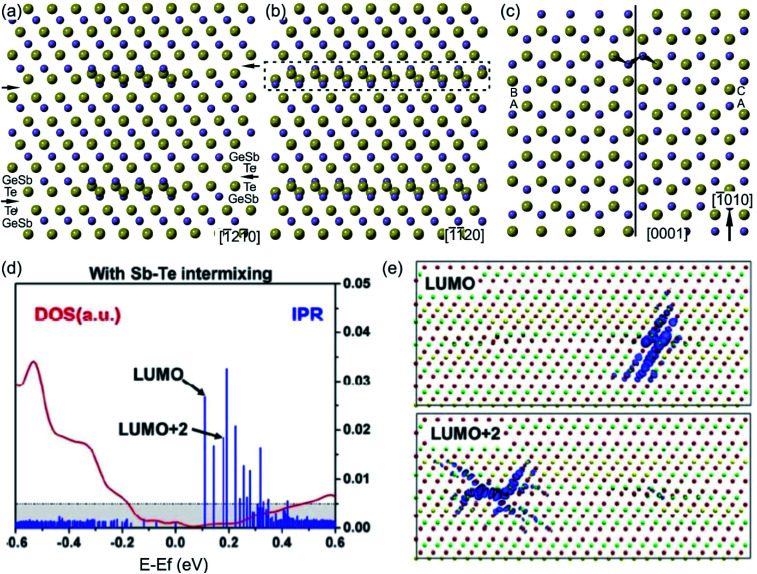
(a)–(c) Unrelaxed structural models of (GeSb)Te bilayer defects. The defects connect GST225 building blocks. Big spheres represent Te atomic columns whereas smaller spheres show mixed GeSb atomic columns. The bilayer defect is highlighted. For the presented zone axis the Te and GeSb atomic columns partially overlap in the defect region. Due to the 3-fold rotational axis, the defect appears every 60° in the 〈11–20〉 zone axis by its rotation around the *c*-axis. However, the lateral width of the same bilayer defect differs in different viewing projections. The arrows in (a) mark vdW gaps. (c) Projection of the (GeSb)Te bilayer defect highlighted by a dashed rectangle in (b) onto the (0001) plane. The continuous line marks a defect boundary between two layers. The layer stacking is AB in the lower part whereas it is AC in the upper part of the image. The lateral width of bilayer defects in (a) and (b) depends on the vertical width of the stacking fault in image (c). The arrows in (c) show snake-like atomic motion of GeSb and Te required for movement of the fault, whereas dashed arrows show the movements necessary for the rearrangement of atoms in one part of the fault, leading to annihilation of the defect. (d) The DOS and inverse participation ratio (IPR), and (e) the corresponding lowest unoccupied molecular orbital (LUMO) and LUMO+2 states. The localization occurs around the bi-layer defects. The empirical threshold IPR value 0.005 that distinguishes localization from delocalization is marked in gray in (d). Adapted with permission from ref. 131. Copyright 2018 American Chemical Society. Notes: (a)–(c) Reprinted from Scripta Materialia, A. Lotnyk, T. Dankwort, I. Hilmi, L. Kienle, B. Rauschenbach, Atomic-scale observation of defects motion in van der Waals layered chalcogenide based materials, 154–158,^130^ Copyright (2019), with permission from Elsevier.

The structural models of bilayer defects viewed along different crystallographic directions are illustrated in Fig. 12(a)–(c).^127^ The defect is confined into two atomic layers of GeSb and Te and represents a localized stacking fault (Fig. 11(c)). The fault is nicely seen by considering the structural model from the [0001] projection. Due to the 3-fold rotational axial symmetry of such GST layered structures, the stacking faults in Fig. 12(a) and (b) belong to different rotation domains. Thus, the fault can appear with different lateral widths in different projections in corresponding atomic-resolution transmission electron microscopy (TEM) images. The widths of the stacking defects in Fig. 12(a) and (b) depend on the length of the fault in the [−1010] direction by looking at defects from the [0001] viewing direction (Fig. 12(c)). In addition, a partial overlap of Ge, Sb and Te atomic columns as well as double positioning of atomic columns at the defect core can be seen from the structural model. This leads to the different occupancy of the Te atom and to the depletion of the image contrast in the defect area in HAADF images. Moreover, a shorter Te–Te distance of 0.26 nm compared to the Te–Te atomic distance of 0.28 nm in the Te metalloid at the fault boundary (Fig. 12(c)) suggests the relaxation of the interface, most probably by a combination of atomic vacancies or Sb–Te antisite defects. The vacancies are particularly favourable for the lateral motion of the defects. On the other hand, the antisite defects influence the total energy of the system and the local electron density.^131^ The bilayer defects increase the total energy of the system and create multiple high energy bands in the tail of the conduction band, resulting in localisation effects (Fig. 12(d) and (e)). The wave functions of these localized states are found around the transition areas of the bilayer defects. However, these electronic states would only be thermally populated at elevated temperatures. In contrast, at room temperatures the bilayer defects do not result in electron localization in the valence band, thus not affecting the metallic state of GST alloys.^131^ Since intensive electron excitations to the conduction band take place at elevated temperatures or under other strong external stimuli, the stacking faults may impact the transport properties of the GST system. In addition, the defects may act as possible sources of electron scattering, affecting the quantitative value of electrical resistance, only.^131^

## 5. Order–disorder transitions and interfacial transformations

### 5.1 Vacancy ordering in phase change materials and cubic–trigonal phase transition

**Fig. 13:**
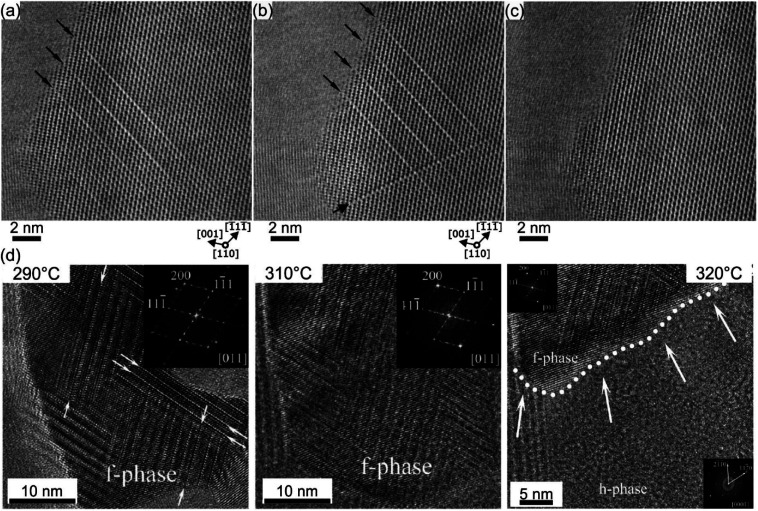
(a)–(c) Atomic-resolution low-angle annular dark-field STEM images of vacancy layers (VLs) produced in the metastable GST225 phase I by focused electron beam exposure of (a) GeSb/V atomic columns and (b) an equivalent 70 inclined lattice plane. (c) The identical specimen position acquired after repeated electron beam exposure of VLs shown in (b). (d) High resolution TEM images acquired during *in situ* TEM heating. The insets show fast-Fourier transform patterns. HRTEM micrographs recorded at a TEM holder temperature of 290° and 310 °C depict grain containing GST225 phase II (marked as the f-phase in the images). HRTEM image acquired at 320 °C shows a grain boundary between phase II and the trigonal phase (marked as h-phases). The images in (d) are reproduced from ref. 134, CC BY 4.0. Note: (a)–(c) Reprinted from Acta Materialia, 105, A. Lotnyk, S. Bernütz, X. Sun, U. Ross, M. Ehrhardt, B. Rauschenbach, Real space imaging of atomic arrangement and vacancy layers ordering in laser crystallized Ge_2_Sb_2_Te_5_ phase change thin film, 1–8,^52^ Copyright (2016), with permission from Elsevier.

Direct observation of atomic scale processes in GST based materials is of particular importance for understanding the fundamentals of phase transitions in these materials. Due to high spatial resolution in direct space, *in situ* atomic-resolution TEM is a method of choice in materials science for direct imaging of dynamic processes in various materials at different length scales. Besides widely used *in situ* heating experiments performed with special TEM heating holders, a focused electron beam in an aberration corrected scanning TEM (STEM) allowing a beam size less than 80 pm can also be applied as a tool for *in situ* manipulation of atomic species in the crystal lattices.^52,127,133^ This enables the study of order–disorder processes in different classes of materials. It was discussed above that the ordering of vacancies into layers is energetically favourable. The ordering process can be indeed induced by electron beam exposure of GeSb/Vs (Fig. 13(a) and (b)).^52^ Due to energy transfer caused by inelastic interactions of the electron beam with the Ge and Sb atomic columns, the Ge and Sb atoms move into the neighbouring cation layers in the 〈111〉 direction. The atoms thus fill empty spaces occupied by vacancies in these cation layers, resulting in the formation of ordered vacancy layers at the end. The process of vacancy ordering leads to the relaxation of the lattice into the newly formed VLs, seen in the shorter Te–Te distance. The transformation of specimen regions with vacancy layers (phase II) back to the initial structure can be achieved by repeated electron beam exposure of the vacancy layers (Fig. 13(c)). These results therefore indicate that the phase transitions between GST225 phase I and phase II are reversible. Thus, this method represents an additional way for a controlled alteration of disorder in the metastable GST225 phases using an electron beam. Moreover, the experimental results on the order–disorder transitions suggest a transformation mechanism between the metastable and stable trigonal GST225 phases. It is proposed that the structural transition to the layered trigonal GST225 phase is driven by the ordering of vacancies in the metastable GST phase.^60^ The ordering process is necessary to occur before complete formation of the vacancy planes. However, atomic-resolution *in situ* TEM heating experiments could not confirm the direct transition from GST225 phase II to the stable trigonal GST225 phase (Fig. 13(d)).^134^ Instead, the grains of vacancy ordered phase II were stable up to transition temperatures to the stable GST225 phase. However, due to the abnormal and very fast grain growth of trigonal GST225 grains at elevated temperatures, a direct observation of the phase transformation was not possible. Although, the exact pathway from the metastable GST225 phase to the stable GST225 phase should be clarified in the future, the transition might include shearing of {111} planes against each other by a vector of a(100)/3 (referring to the trigonal phase), similar to martensitic transformation processes.^135,136^

### 5.2 Interlayer exchanges in layered Ge–Sb–Te crystal structures

**Fig. 14:**
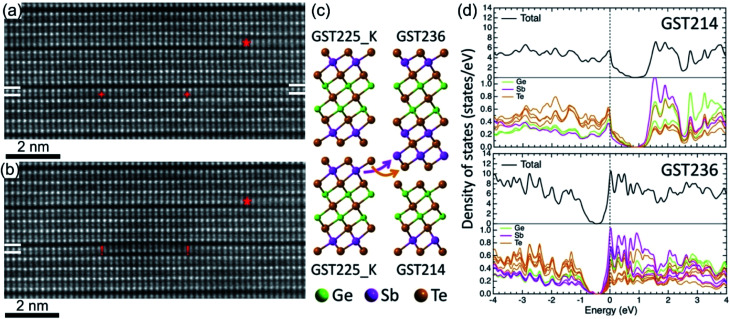
*In situ* imaging of GeSb and Te atomic layer reconfiguration in the GST225 crystal structure under the influence of focused electron beam irradiation. Atomic-resolution HAADFSTEM images of the (a) initial state and (b) after reconfiguration of (GeSb)Te bilayers within the GST225 lattice with subsequent formation of new vdW gaps. Due to incoherent imaging (Z-contrast or HAADF), the intense dots in (a) and (b) correspond to Te atomic columns, whereas the less intense dots represent GeSb atomic columns. Dark lines are from vdW gaps. The marks “+” show the position of the electron beam scanning line, while the marks “!” mark the areas with bilayer stacking faults. The mark “*” depicts a pre-existing bilayer defect for visual guidance. (c) Schematic representation of the decomposition of GST225 blocks into GST214 and GST236 building units. (d) The calculated electronic densities of states for GST214 and GST236 phases. The Fermi energy level is set to 0 eV. Reprinted from ref. 139. With the permission of AIP Publishing. Notes: (a) and (b) Reprinted from Acta Materialia, 141, A. Lotnyk, U. Ross, T. Dankwort, I. Hilmi, L. Kienle, B. Rauschenbach, Atomic structure and dynamic reconfiguration of layered defects in van der Waals layered Ge–Sb–Te based materials, 92–96,^127^ Copyright (2017), with permission from Elsevier.

The vdW gaps in stable trigonal GST crystal structures represent empty spaces between the adjacent Te layers, thus representing vacancy layers similar to GST225 phase II. Consequently, similar processes of atomic-scale motion towards the vdW gaps are assumed. As expected, focused electron beam exposure of GeSb layers next to vdW gaps within a GST building block (Fig. 14(a)) results in the reconfiguration of (GeSb)Te bilayers with subsequent formation of new vdW gaps (Fig. 14(b)).^127,137^ The adjacent building blocks reconfigure locally into 4-fold Te and 5-fold Te layered structures, resulting simultaneously in the formation of a (GeSb)Te bilayer stacking fault. Depending on the beam fluence, the newly formed vdW gap can either rearrange back to the initial state or move in the lateral direction along the vdW interface.^127,137^ In addition, the reconfiguration process of vdW gaps leads to the local change of stoichiometry of individual GST building units. As a consequence, the cation layers of the newly produced 4-fold Te layered structure next to the artificially produced vdW gap become Ge-rich instead of Sb-rich. This change in local composition impacts the electronic properties of the GST materials. First-principles calculations demonstrate that changing the local stoichiometry of GST building units results in a finite density of states at the Fermi level and leads to the elimination of the band gap.^132,138^ However, further electronic density of states (DOSs) simulations show that such non-stoichiometric local structures (Fig. 14(c)) possess a narrow bandgap of about 0.2–0.3 eV.^139^ Depending on the local composition of the cation layers close to the vdW gaps, the Fermi level of such GST structures can be located either in the valence or in the conduction band, suggesting p-type (GST214 in Fig. 14(d)) and n-type (GST236 in Fig. 14(d)) semiconductors. In addition, electron counting analysis for such building blocks suggests electron deficiency for the GST214 block and electron excess for the GST236 unit, while the electron balance of the GST225 unit cell is neutral.^139^ Thus, the results are in line with the DOS simulations. Although theoretical calculations still consider GST structure models with ordered cation layers, the theoretical results offer a new approach for material design of vdW bonded compounds by tuning the local stoichiometry of the compounds at the vdW interfaces. Moreover, the experimental results (Fig. 14(a) and (b)) and simulations are also of high importance to understand the switching mechanisms in layered GeTe–Sb_2_Te_3_ based heterostructures.^74,138–141^

### 5.3 Ultrafast interface-controlled crystallisation processes

In a PCM device, one of the most performance relevant aspects is the memory writing time, which is mainly limited by the SET process involving the crystallization of the phase change material. However, being an intrinsic feature of the material, the crystallization speed is difficult to push beyond a certain limit. Theoretical simulations predict crystal growth velocities in the range of 0.7–1.5 m s^−1^,^142–146^ whereas ultrafast differential scanning calorimetry measurements give larger values for the growth velocity (2.5–3 m s^−1^).^25^ In order to enhance the crystallisation speed, several strategies have been developed, including doping of the material or using GST based heterostructures.^14,106,147–149^

**Fig. 15:**
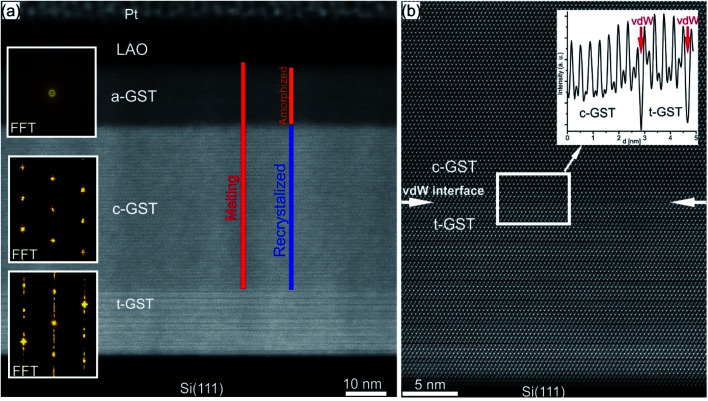
(a) HAADF-STEM image of an epitaxial t-GST thin film after irradiation with a single ns-laser pulse. Three distinct regions are identified and corresponding FFT patterns are inserted on the left-hand side. At the bottom of the thin film, the initial structure of epitaxial t-GST remained, whereas in the middle region, a layer of c-GST formed. At the top of the thin film, the GST material quenched into the amorphous phase. (b) HAADF-HRSTEM image of the interface between the remaining t-GST structure and the recrystallized c-GST structure (indicated by red arrows), revealing an epitaxial relationship between the two layers. In addition, an intensity profile across the interface is shown in the top right corner. Reproduced from ref. 83 with permission from the Royal Society of Chemistry.

Recently, a promising approach to study the crystallization capabilities of GST225 has been presented based on interface-assisted epitaxial recrystallization of GST225 phase I (c-GST225) in epitaxially grown GST225 thin films.^83^ In detail, single ns-laser pulse irradiation of an epitaxial trigonal GST225 (t-GST225) thin film caused partial melting of the film followed by ultrafast recrystallization during the cool down process. Fig. 15(a) shows HAADF-STEM micrographs of the t-GST225 thin film after single ns-laser pulse irradiation. The cross-section of the thin film (Fig. 15(a)) can be divided into three distinct regions. At the bottom of the thin film the initial t-GST225 structure remains, whereas in the middle and at the top a-GST225 and c-GST255 form, respectively. Interestingly, an epitaxial relationship between the t-GST225 and the c-GST225 layer is observed (Fig. 15(b)), indicating the recrystallization of c-GST225 from the transient molten phase. The formation of the cubic structure is determined by crystal growth, starting at the remaining planar t-GST225 template and proceeding in an epitaxial fashion towards the top of the thin film. However, due to the higher cooling rates towards the top of the GST thin film, the propagating crystallization front, separating between the solid and liquid phase, changes into an amorphization front once the critical cooling rate for amorphization is passed. In essence, the results demonstrate ultrafast epitaxial crystal growth of c-GST225 at high temperatures providing on the one hand access to crystal growth kinetics of GST225 materials in the ultrafast regime and on the other hand a possibility to increase memory writing times by bypassing the incubation period usually occurring during nucleation dominated crystallization of GST. Owing to the capability to speed up the switching process, this interfacial assisted crystal growth was also suggested for nanometric volumes of GST by advanced computer simulations underlining its potential.^142,150^

In the future, the interface-controlled approach can be used to study the mechanisms of phase transitions in a wide variety of similar structures. The combination with time-resolved experiments will allow the quantification of crystal growth velocities and the determination of crystallization limits of GST based materials, necessary for designing new alloys for memory applications.

## 6. Conclusions

Ge–Sb–Te based phase change alloys represent an outstanding class of functional materials having a tremendous variety of industrially relevant properties. In recent years, the phase change memory based on these alloys has become an emerging technology enabling to advance the performance of existing memory devices. Many efforts have been devoted to the understanding of material properties with the particular aim to develop a universal memory. Thus, it is important to comprehend the relevant properties of Ge–Sb–Te based phase change materials, which have been addressed in this review. Particular focus was made on atomic structure, impact of disorder on material properties, order–disorder transitions and interfacial transformations.

At the beginning, this paper elucidates the complex structural data of various crystalline phases of ternary Ge–Sb–Te chalcogenide-based alloys. According to the current state of knowledge, there are several modifications of the cubic metastable phase due to a huge number of structural vacancies which can obey different distributions: they can be either randomly distributed (phase I) or arranged into layers (vacancy layers, phase II). The stable trigonal Ge–Sb–Te phases also show a highly ordered distribution of vacancies. However, the vacancy planes form so-called van der Waals gaps. All phases are distorted and disordered on the cation sublattice. The difference between metastable vacancy ordered phase II and the stable trigonal phase is in distinct stacking sequences of adjacent Te layers. For the metastable phases, the Te–Te stacking across the vacancy layer is cubic, whereas the stable phases exhibit a hexagonal type stacking of Te–Te layers across a van der Waals gap. Moreover, the Te–Te distances are relaxed into the vdW gap. In addition, phase II and the stable trigonal phase can be viewed as layered structures.

The spatial distribution of vacancies in crystalline Ge–Sb–Te lattices has a large impact on the electronic and optical properties of crystalline Ge–Sb–Te materials. The phases with ordered vacancy layers (phase II) show metallic behaviour, while Ge–Sb–Te phases with disordered vacancies possess insulating behavior. In addition, vacancy ordered phases have larger reflectivity compared to vacancy disordered phases. These phenomena are attributed to electron localisation effects. Since the structural order in crystalline Ge–Sb–Te materials correlates with different resistivity states, offering much stable resistance states over time, control of disorder in such materials is very important for applications. It provides another route to enable multi-level data storage. In addition to thermal heating, laser irradiation and targeted electron beam irradiation, precise control of disorder in such materials can be achieved by epitaxial growth of Ge–Sb–Te thin films with single-crystalline quality since it allows growth of thin films with specific arrangement of vacancies. However, stable trigonal Ge–Sb–Te thin films possess chemical disorder, leading to the formation of building units with different local stoichiometries. Characteristic structural defects in such layered Ge–Sb–Te crystal structures include rotational twin domains and particular bilayer stacking faults. The bilayer defects result from structural reconfiguration of vdW interfaces in layered Ge–Sb–Te compounds and usually connect building units of different local stoichiometries. This local alteration influences the electronic properties of Ge–Sb–Te materials, *e.g.* a finite density of states at the Fermi level or change of the Fermi level of such GST structures resulting in a p or n conductor. Moreover, the bilayer defects may affect the transport properties of Ge–Sb–Te based systems, resulting in localized wave functions at elevated temperature. However, the defects do not affect the metallic state of phase change alloys at room and low temperatures. The importance of bilayer defects and structural reconfiguration of vdW interfaces can be additionally found in the debate on the switching mechanism of GeTe–Sb_2_Te_3_ based superlattices.

The current concept of multilevel data storage is based on controlling the ratio between amorphous and crystalline Ge–Sb–Te alloys. However, the concept reaches its limit when the memory cell is scaled down to a few nanometres.^151^ Thus, very precise control of the amorphous–crystalline volume ratio of PCM cells is very important. This can be achieved by using epitaxial Ge–Sb–Te thin films. Due to interface assisted crystal growth along preferred crystallographic directions, the volume ratio can be precisely controlled. In addition, this approach will significantly enhance the crystallization speed of Ge–Sb–Te materials, thus improving one of the main drawbacks of PCM, namely memory writing speed.

The use of highly textured GeTe–Sb_2_Te_3_ based heterostructures is one promising approach for further engendering of PCM devices with low switching energy and high switching speed.^147^ Although the proof-of-concept of such devices meets the main requirements of semiconductor technology, memory devices based on epitaxial single-phase Ge–Sb–Te materials are not employed yet. Since the growth of highly crystalline epitaxial thin films of Ge–Sb–Te materials can be realised much easier compared to the superlattices, resistive switching characteristics of epitaxial single phase thin films and nanostructures will present an outlook for future opportunities of chalcogenide based alloys in PCM technology. The influence of the density of bilayer defects, crystal structure and degree of disorder on the switching characteristics of such PCM devices should be identified in the near future too.

## Conflicts of interest

There are no conflicts to declare.

## Supplementary Material
